# Clinical educators’ experiences of facilitating learning when speaking a different language from both the student and client

**DOI:** 10.1186/s13104-017-2874-4

**Published:** 2017-11-02

**Authors:** Nicola Keeton, Harsha Kathard, Shajila Singh

**Affiliations:** University of Cape Town, Faculty of Health Sciences, F56 Old Main Building, Groote Schuur Hospital, Observatory, Cape Town, Western Cape 7925 South Africa

**Keywords:** Multilingual, Clinical education, Diversity, Assessment

## Abstract

**Background:**

Worldwide there is an increasing responsibility for clinical educators to help students from different language backgrounds to develop the necessary skills to provide health care services to a linguistically diverse client base. This study describes the experiences of clinical educators who facilitate learning in contexts where they are not familiar with the language spoken between students and their clients. A part of the qualitative component of a larger mixed methods study is the focus of this paper. Semi-structured interviews were conducted with eight participants recruited from all audiology university programmes in South Africa. Thematic analysis allowed for an in depth exploration of the research question. Member checking was used to enhance credibility. It is hoped that the findings will inform training programmes and in so doing, optimize the learning of diverse students who may better be able to provide appropriate services to the linguistically diverse population they serve.

**Results:**

Participants experienced challenges with fair assessment of students and with ensuring appropriate client care when they were unable to speak the language shared between the client and the student. In the absence of formal guidelines, clinical educators developed unique coping strategies that they used on a case-by-case basis to assess students and ensure adequate client management when they experienced such language barriers while supervising. Coping strategies included engaging other students as interpreters, having students role-play parts of a session in English in advance and requesting real-time translations from the student during the session. They expressed concern about the fairness and efficacy of the coping strategies used.

**Conclusions:**

While clinical educators use unique strategies to assess students and to ensure suitable client care, dilemmas remain regarding the fairness of assessment and the ability to ensure the quality of client care.

**Electronic supplementary material:**

The online version of this article (10.1186/s13104-017-2874-4) contains supplementary material, which is available to authorized users.

## Background

In Australia, the USA and South Africa, larger numbers of diverse clients requiring linguistically appropriate services has resulted in a greater demand for universities to train professionals from different language backgrounds [1, 2]. In South Africa, Higher Education institutions continue to be responsive to the call for redress and an increasing number of students from different language groups [3] are entering the Health Sciences programmes for training. Clinical education is a core element of Health Sciences training programmes and clinical educators are now responsible for helping students from different language backgrounds to develop the necessary skills to provide services to a linguistically diverse client base. Supervision includes the observation, facilitation of acquisition of knowledge and skills, guidance and assessment of any student–client interaction, and may occur in a language not spoken by the clinical educator. While in developing countries interpreters are often used to manage language barriers between clients and student clinicians [4], interpreters are scarce in the South African public sector due to financial restrictions [5, 6]. As more students are able to provide services to clients in their mother tongue, clinical educators must ensure that assessments are fair and that students provide quality care. The provision of clinical services in a language spoken by a student and not the clinical educator can be challenging for all parties involved [2, 7, 8].

Clinical educators often have a dual responsibility; as the clinician they are in charge of client care but equal responsibility is assumed for providing appropriate learning opportunities for students [9, 10]. In an opinion piece, Muñoz et al. [7] suggest that clinical educators in the USA may feel that they are unable to adequately meet the needs of the client or the student when they are unable to understand the content of a session. Additionally, students may have misgivings about their learning experiences and the appropriate management of clients when their supervisor is unable to understand the student–client dialogue [2]. Verdinelli and Biever [2] qualitatively examined the perspectives of fifteen Spanish-speaking psychology students in the USA who were required to provide services to a Latina population while supervised by clinical educators who did not speak the language. Participants felt that clinical educators were unable to provide an objective opinion on the finer points of their sessions and tended to provide more thorough input on sessions in English. Findings suggest that while clinical educators were attempting to provide appropriate supervision in this challenging environment, they fell short and ultimately the training did not adequately prepare students to provide services to a Spanish-speaking population.

An additional challenge for clinical educators is that of providing reliable and valid assessment of sessions that occur in a language that is not understood. Clinical educators are required to assess both a student’s clinical knowledge and skills as well as their generic abilities such as interpersonal skills, communication and professionalism [11]. The ability to assess multicultural clinical sessions fairly and objectively has been highlighted by authors in the USA as a specific challenge [12]. Assessment where the clinical educator does not speak the language of the student clinician and their client may have additional challenges and there is insufficient research examining how these sessions are evaluated.

While international literature [2, 7, 8] has described the complexity of facilitating clinical learning in the absence of understanding content and has examined the experiences of bilingual students, research in the area of clinical education and language barriers is limited. No framework has yet been proposed for the facilitation of clinical learning when students are providing services in a language not spoken by the clinical educator. With Health Sciences programmes emphasizing the importance of training linguistically diverse students who might provide appropriate services to clients from diverse language backgrounds it becomes imperative to examine current practices with a view to creating models to support training for a new generation of health professionals.

Findings reported in this paper were generated from a larger mixed methods study examining clinical educator experiences and perceptions of working with students from diverse race and language backgrounds as part of a thesis [13]. The full dissertation was published in a book [14] and while it explains that clinical educators experienced challenges and used coping strategies when working with students from diverse language backgrounds, it does not discuss findings related to this article’s topic in any detail.

This manuscript is an extension of the thesis and aims to describe the experiences of clinical educators facilitating learning and assessing students providing services to a linguistically diverse population. Additionally, it examines the coping strategies that clinical educators in South Africa are currently employing in order to assess students and to ensure that quality client care is provided when the student–client interactions occur in a language they are unfamiliar with. While this study examines the experiences of audiologists, it is felt that the clinical education process is similar for most health professionals so that findings could be applied to Physiotherapy, Occupational Therapy, Speech Language Therapy, Medicine and Nursing.

## Methods

Approval to conduct the study was obtained from the Faculty of Health Sciences Human Research Ethics Committee at the University of Cape Town (424/2010). Purposeful criterion sampling [15] was used to select eight clinical educators who had completed questionnaires (sent to all known Audiology clinical educators in South Africa) in the initial quantitative phase of the original study [13]. Polit and Beck [15] suggest that participants with specific differences but who have shared a common experience should be selected in order for the researcher to explore all aspects of the phenomenon under investigation to increase the richness of data and to allow for transferability of findings. Participants who represented the greatest variety of viewpoints, based on questionnaire responses from the original study, were deemed to be information rich and selected to achieve data saturation [15]. Table 1 provides a description of participant demographics. All participants selected for the interviews were female and spoke English proficiently. Participants were selected to be most representative of the Audiology clinical educator population at the time. While approximately 9.6% of the South African population report English to be their first language [16], the majority of audiologists working in the country are only able to provide services in English or Afrikaans [6]. Participants had all completed at least a full year obligatory course in one of the African languages that make up the eleven official South African languages as part of their degree.

**Table 1:** Participant demographics

Race	Black	Indian	Coloured	White
	n = 1	n = 1	n = 2	n = 4
Mother tongue	English	Afrikaans	Sesotho	
	n = 4	n = 3	n = 1	
Employer	University	Clinical site		
	n = 4	n = 4		

Race was self-declared and categorized according to each of the four predominant population groups as described by the South African Statistics association (2009): Black; Coloured; Indian; White

Clinical educators working for the university as well as those working at clinical sites were included. While participants supervised in different areas of clinical practice they all supervised through direct observation of a student–client session and assessed using mark sheets that evaluate clinic-specific knowledge and skills, clinical reasoning, client assessment and management, communication and/or written work.

Informed consent was obtained prior to conducting an hour and a half semi-structured interview with each participant. The open-ended nature of questions in the interview schedule explored participants’ expectations of clinical performance based on a students’ race and language proficiency level, and their experiences of teaching, managing and assessing students from diverse race and language backgrounds (see the interview schedule in Additional file 1). All eight interviews discussed the issue of providing supervision to students when the clinical educator does not understand the language used in the session.

Elements from Colaizzi’s steps for thematic data analysis as described by Sanders [17] were incorporated to analyse the data. The researcher started by familiarisation and immersion with the transcriptions. The data was then coded and themes were created and elaborated on. Data saturation was achieved when no new themes could be identified from the data. Finally, member checks were conducted twice to improve rigour by enhancing the credibility and trustworthiness of reported findings. First the analysed data in themes and then a draft of the results and discussion section of the dissertation were emailed to participants. They were asked to comment on the accuracy of the analysed data and to expand on or clairfy any issues [15]. Both times, all participants responded that no changes to the analysed data or to the discussion of quotes used in the study were necessary.

## Results

The analysis revealed two major themes (namely: *challenges* and *coping strategies*) that described clinical educators’ experiences when supervising a session conducted in an African language that was not understood by the participant. The first theme detailed the *challenges* experienced when attempting to ensure appropriate client care and when assessing the student while the second described *coping strategies* the participants used to overcome them.

### Challenge: client care

While the participants noted the value in students being able to provide services in the client’s mother tongue, seven reported that it was challenging to ensure that the client’s needs were appropriately and completely addressed when the content of the session was not understood. It was also a concern that the client might not accurately comprehend important information (5 participants). If the student did not recognize the error or did not relate this information to the clinical educator due to fears of losing marks, the client would leave without crucial feedback or management information.

“…. you don’t always know that the student has told you properly what was said. At the end of the day, I am responsible for the patient and we have had experiences where the patient has not understood the feedback which made them really distressed. But… you have no choice, you have to trust the student and just do whatever you can to make the best of the situation.”

Participants noted that the relationship between themselves and the student relied on a high degree of trust in order to ensure effective client care. Three participants made a suggestion that this trusting relationship could be challenged, as students might not be completely honest if they believed that sharing what they had or had not told the client might result in a lower mark.

“We are ultimately responsible for the patients’ care and… It is difficult because you rely heavily on the student to be honest in telling you what they have done and they aren’t always honest [when they are scared of being marked down].”

### Challenge: assessment

Seven participants reported that it was challenging to accurately assess a student’s performance when they were unable to understand the session content because they did not speak the language used by the student and client.

“I understand a little bit of for instance, Tswana so I would be able to follow a conversation and the general gist of what a student is doing… but I still don’t think you can get an accurate view of their skills if you don’t understand or it’s not translated back to you…”

Six participants were especially concerned about the accuracy of their marking. Evaluating the session and allocating marks became challenging as while certain improvements in the students’ performance could be observed (such as body language and the development of rapport with the client) the participants were not certain what content had been relayed to the client.

“The interesting thing is that their [the students’] whole demeanour changes when they’re able to converse with the patient in their own language. It presents challenges as you don’t understand necessarily what they’re saying but they are … more confident and the patient is able to relate to them more easily… I can give them better marks for communication… You shouldn’t give them the marks if you don’t know what they are saying, though…”

Three participants were unsure how to use the mark sheet in these situations and each marking session where language barriers occurred was assessed differently. The fairness, integrity and reliability of the assessment were questioned as participants noted that by uniquely adjusting student marks, uniformity of the assessment process was affected.

“[I leave out sections that could not be marked because content was not understood] but I don’t know that that’s fair either because other students may lose marks on those sections and now this student gets a better mark because I didn’t understand what they said…”

“Its not really fair but I can’t always fill out all the necessary marks because I didn’t understand what was said… the marks are not really a true reflection of the student’s performance when I compare them with other students.”

There was a definite sense of frustration at the difficulty of having to assess sessions where content was not understood.

“It is very difficult to assess when you yourself do not know the language they are speaking… It’s frustrating.”

### Coping strategies

Participants detailed various coping strategies they were using in order to ensure optimal client care and to appropriately assess and manage sessions where the content was not understood. While most of the strategies were consistent with the literature, there were a few novel strategies described by participants. The most common strategies reported by participants have also been proposed by other authors on the subject and include the:

- Provision of real-time explanations/translations by the student during the session as required [2, 7] (7 participants).

“I like to ask my students to explain what the client is asking them [the student] during feedback so I know if I need to step in and guide the session more. If I do then the student will have to interpret for me… I need to know that the patient has had all their questions answered…”

“…I then mark based on what the student tells me she is saying to her patient.”

- Allocation of additional time to discuss cases [7] (6 participants).

“I will often need extra time to talk through the cases, to make sure that I understand what happened during the session which can be difficult in a busy clinic….”

- Careful observation of interpersonal skills and metalinguistic communication used to develop the student–client relationship [7] (4 participants).

“You need to watch the session carefully and pay special attention to [the student’s] interpersonal skills and take note of the relationship they are building with the patient.”

“It is important to watch the patient to see if they look confused. If they are nodding and looking interested then it is usually fine…”

The following unique coping strategies emerged and to our authors’ knowledge have not been reported in the literature.

Other students are asked to “*translate*” during the session (5 participants).

“If another student speaks that language then I ask them to ‘interpret’ for me during the session. It is easier for me to ensure proper patient care if I understand what the student is saying.”

Students role-play feedback with another student in English prior to engagement with the client to allow for assessment of content (4 participants).

“The students need to role-play the feedback with another student in English so that I can assess exactly what they’re gonna say. That’s when I give them marks”

“Role-plays are great—you can get a feeling that the student understands exactly what they need to cover … and guide them if they’re missing any important management [decisions].”

Students are required to submit written case history questions in English before the session (4 participants).

“I … give a mark for case history based on the written questions they have brought me. I will then also obviously have to try and check that they do actually use what they have written with the client by asking questions in the session.”

Clinical educators guess content based on knowledge of basic vocabulary in the language and careful observation (3 participants).

“I do have some basic knowledge of the languages so I usually just mark like normal and then guess what is being said. You can see if the patient is following what is being said and you can watch the testing to make sure that the patient is doing what is needed.”

Clinical educators leave out sections assessing communication on the mark sheets (3 participants).

“Sometimes I just adjust the mark sheet and leave out certain marks like for the bits I didn’t really understand ….. like the bits about whether they could manage a communication breakdown or clarify information or even if they showed empathy or gave instructions clearly…..”

## Discussion

Participants used various coping strategies in an ad hoc manner and reported a number of challenges when supervising students where the language being spoken was not understood. Concerns were expressed about the efficacy and accuracy of these measures to ensure valid and reliable assessment of students and to guarantee optimal client care. All, except one participant, were English or Afrikaans speaking South African clinical educators. The clinical education context is one where students need to provide linguistically appropriate services to the previously neglected majority population who mostly speak African languages. The national drive has been to recruit linguistically diverse students into the Audiology training programmes to provide comprehensive care to clients in their home language. In some instances, for student assessment purposes, clinical educators might ask students to attempt a session in rudimentary English or Afrikaans if the client speaks these second languages. The decision is unpopular, as clients may not understand what is being communicated. The participants were thus struggling to negotiate decisions for client care and student assessment where language formed a barrier to understanding the session content.

### Client care

Participants reported that they found it challenging to ensure that the student was appropriately meeting client needs. It became evident that many coping strategies that were used to ensure optimal client care relied on a high level of trust between the clinical educator and the student. Some strategies used by participants had also been reported by Muñoz et al. [7] (such as ensuring the educator was in the therapy room so that students could give real-time interpretations allowing for immediate adjustments to client care). These strategies rely on the student’s ability to clearly and honestly relate what was discussed with the client. The participants’ concerns may be valid as a student might feel the need to adjust the information they give to the clinical educator in fear of losing marks although the participants did not give any evidence to support this claim. Verdinelli and Biever [2] suggest that students may simply find it difficult to translate clinical language into the client’s mother tongue. While their study examined the perceptions of Spanish-speaking students, it can be suggested that many clinical terms or therapeutic concepts may also be difficult to translate into African languages making it difficult for a student to ensure that their client comprehensively understands the pertinent information. Participants may have been unaware of challenges their students face when attempting to relate what has been discussed with a client. Clinical educators may need to learn how to create a trusting relationship between themselves and their linguistically diverse students where both might share their concerns and challenges in a way that provides optimal client care.

In this study, participants reported some novel coping strategies for ensuring client care that authors could not find to be described in the literature. For instance, a few participants would, where possible, ask another student to interpret what was occurring in the session. While this strategy gives the clinical educator access to session content, it again relies on the honesty of a student and the ability of that student to accurately relay session content. It might be suggested that students could feel uncomfortable relating information that their friend is giving the client that they know is incorrect as they may not want their peer to be penalized for mistakes. Verdinelli and Biever [2] suggest that students might also feel that it is inappropriate to be used as an interpreter (between the clinician and client) instead of as a clinician. Clinical educators would need to negotiate with each student to ensure that they are comfortable taking on the role of an interpreter and should highlight the importance of this role in the learning journey of their peer.

Many of the participants reported that they often use role-play or ask students to write down the exact information that they plan to share with their clients. To the authors’ knowledge, these strategies have not been discussed elsewhere in the literature. While some content might change slightly when translated into a different language, assessment of role-play or written content to evaluate student understanding of what they should tell a client and the manner in which to do so, may make clinical educators feel more confident that the essential needs of the client will be met.

In attempting to cope with these language barriers, the participants put mechanisms in place to support student learning and to ensure client care. Participants were creating strategies independently and managing each situation differently.

### Assessment

There were differing perceptions of the efficacy, reliability and/or fairness of the reported assessment strategies adopted. Adjusting mark sheets to assess only those parts of the session that could be followed by clinical educators has not been previously reported. As more multilingual students enter Health Sciences professions, there is a greater need to ensure that there is a regulated means for fair assessment of students providing linguistically appropriate services. Development and use of different ways to assess performance when there was no understanding of the content calls into question the fairness and reliability of these methods.

For instance, participants were concerned that students sometimes achieved better marks for client interaction in these sessions than they would have if the clinical educator could understand the content. The participants reported that improved non-verbal student–client communication was perceived, but wondered whether the mark accurately reflected the level of performance, as they could not understand the content. While there is an advantage for service provision in a client’s first language, assessment using only non-verbal cues and student reports of the communication may not be reliable. If the assessment is not a true reflection of a student’s performance, the student will not be aware of areas that require improvement. Except for the article written by Muñoz et al. [7], the authors could find no literature discussing the assessment of sessions where content is not understood by the clinical educator and it may be suggested that uniquely modified assessments could unfairly advantage or disadvantage students. This study therefore throws light on a new dimension of challenge that could not be found to be reported on in the literature.

There is a noticeable lack of research providing insight into appropriate assessment practices to be used when assessing performance where language barriers occur in clinical education. Muñoz et al. [7] suggested that standard assessment processes should be modified, but details on how this should be accomplished need further exploration.

In all of the examples discussed, participants were modifying their management of students as well as their assessment of sessions by trial and error. This study highlighted some innovative strategies participants were using to train students in a complex clinical learning environment but their dilemmas suggest that further research is needed to develop guidelines for supervision and assessment of students in linguistically diverse environments.

## Study limitations

The small sample taken from an Audiology background in South Africa suggests that caution should be exercised in generalizing the findings to other countries and disciplines. Participants were selected to be most representative of the Audiology clinical educator population in South Africa in order to achieve triangulation and to provide a comprehensive understanding of the different experiences across the country. Clinical educators in more developed countries and in other disciplines may have different challenges.

## Study implications

While a number of challenges regarding the training of multilingual Health Sciences students have been outlined in this paper, findings suggest that the first steps have been taken towards identifying issues and describing strategies that support better training of diverse students providing services to clients speaking different languages and so have relevant implications for future practice. Two areas in Audiology education programmes require review and modification to ensure that multilingual students receive the necessary support to provide effective services to linguistically diverse clients. Firstly, changes need to be made within the curricula to facilitate appropriate learning opportunities and secondly, clinical educators need to be trained.

### Curricula

Clinical curricula need to be regularly evaluated to investigate whether the expected learning outcomes regarding provision of services to multilingual clients are appropriately aligned with teaching and learning activities as well as assessment measures [12]. Mark sheets should reflect the importance of appropriate service provision to diverse clients in order for programmes to comply with health profession’s regulations for curricula. Evaluation of this alignment will help to identify strengths and weaknesses within the system that will aid programmes to improve their ability to train culturally competent clinicians [12].

### Professional development

While this study highlighted that participants were unprepared for the challenges of clinical education in a linguistically diverse training environment, it is positive that the clinical educators were creative in finding ways to support students. Training programs specifically targeting the assessment and management of sessions occurring in a language not understood by the clinical educator would provide the necessary support to clinical educators training a new generation of multilingual professionals.

## Conclusions

Training multilingual students to provide services to a linguistically diverse client base can be challenging especially for monolingual clinical educators who are unfamiliar with these languages. The lack of uniformity in terms of how to fairly assess students where language barriers exist in clinics as well as how to provide appropriate models of service delivery to linguistically diverse clients suggests the need for guidelines and/or protocols to be implemented. This study provided insight into clinical educator experiences that may determine interventions to facilitate the clinical education process in this context and support the development of guidelines to contribute to training that optimizes the learning of all students. Effective training of students from different backgrounds might ultimately help to graduate professionals who will best be able to provide linguistically appropriate health care to the diverse South African population.

## Additional file

**Additional file 1.** Interview schedule.
