# Supplementary material for: Clinical educators’ experiences of facilitating learning when speaking a different language from both the student and client
Source: BMC Res Notes. 2017 Nov 2;10:546. doi: 10.1186/s13104-017-2874-4 (PMC5667452; doi:10.1186/s13104-017-2874-4)
Supplement: Supplementary file 1 — Additional file 1. Interview schedule. [file 13104_2017_2874_MOESM1_ESM.docx]

**Additional file 1**

**Interview Schedule**

My research is looking at Clinical Educators’ experiences and expectations that arise from working with students from different race and language groups in clinics. Facilitating learning in clinics poses a number of challenges and unique situations for us, especially now that the patients our students see are from such diverse backgrounds. In this interview I would like you to take time to share your thoughts with me about supervising in this environment.

Firstly, what clinics are you responsible for supervising?

1. Describe some of your general experiences of supervising students from diverse backgrounds within a clinical setting.
   - PROBE:
     - Student backgrounds
     - Specific examples
     - Challenges vs Rewards
2. Tell me about the clinical performance of students from diverse race and/or language groups.
   - PROBE:
     - Contributing factors (Schooling, Language, *Culture, resources)
     - Similarities vs differences
3. Describe how you teach clinical skills to students from diverse race and/or language backgrounds.
   - PROBE:
   - *Do you feel adequately prepared to supervise? In what ways did the supervision course you attended at the university help you to deal with the challenges you experience as a clinical educator -diversity?*
     - Patient vs student needs
4. Describe your experience in assessing students from diverse backgrounds in a clinical setting.
   - - Tell me about challenges and/or rewarding experiences
     - Influencing factors
5. In what ways have your experiences shaped your expectations of the clinical performance of students from diverse race and/or language backgrounds?
6. Is there anything else you would like to share with me?
